# Supplementary material for: Large-scale cross-ancestry genome-wide meta-analysis of serum urate
Source: Nat Commun. 2024 Apr 24;15:3441. doi: 10.1038/s41467-024-47805-4 (PMC11043400; doi:10.1038/s41467-024-47805-4)
Supplement: Supplementary file 1 — Supplementary Information [file 41467_2024_47805_MOESM1_ESM.pdf]

Supplementary Information

Supplementary Fig. 1. Mirrored Manhattan plots of GWAS and TWAS in the European and East Asian ancestry meta-analyses……………………………………………………………………………….…2

Supplementary Fig. 2 Comparison of effect sizes for the GWAS using UKBB-provided PCs and the GWAS using newly calculated PCs in UKBB European…………………………………………….3

Supplementary Fig. 3. Comparison of effect sizes for the GWAS using UKBB-provided PCs and the GWAS using newly calculated PCs in UKBB non-European…………………………………………...4

Supplementary Fig. 4 Comparison of the effect size and direction of the lead variants in the cross-ancestry meta-analysis with those in the GWAS of each UKBB non-European population……………..5

Supplementary Fig. 5 Regional plots of 17 previously unreported significant loci in the cross-ancestry meta-analysis……………………………………………………………………………………6

Supplementary Fig. 6 Genetic correlations between traits according to ancestry……………………….9

Supplementary Fig. 7 Functional enrichment analysis in the European and East Asian ancestry meta-analyses…………………………………………………………………………………………..10

Supplementary Fig. 8 Leave-one-out polygenic risk score (LOO PRS)……………………………..11

Supplementary Fig. 9 PRS phenome-wide association study (PheWAS) plot applying the PRS of European ancestry to the European population (UKBB)……………………………………………12

Supplementary Fig. 10 Kaplan-Meier survival curves for gout, heart failure, and essential hypertension with the cross-ancestry and European ancestry PRS…………………………………………………...13

Supplementary Fig. 11 Comparison of disease prevalence according to PRS ancestry in the Korean population……………..……………………………………………………………………14

Supplementary Fig. 12 Comparison of odds ratios by PRS ancestry in the Korean population………15

Supplementary Fig. 13. Comparison of ROC curves of the PRS of the East Asian ancestry in the Korean population……………………………………………………………………………………..16

Supplementary Fig. 14 Comparison of ROC curves by cross-ancestral PRS in the Korean population..17

URLS…………………………………………………………………………………………18

Supplementary Fig. 1 Mirrored Manhattan plots of GWAS and TWAS in the European and East Asian ancestry meta-analyses

(a) GWAS of European ancestry and TWAS. (b) GWAS of East Asian ancestry and TWAS. GWAS, genome-wide association study; TWAS, transcriptome-wide association study.

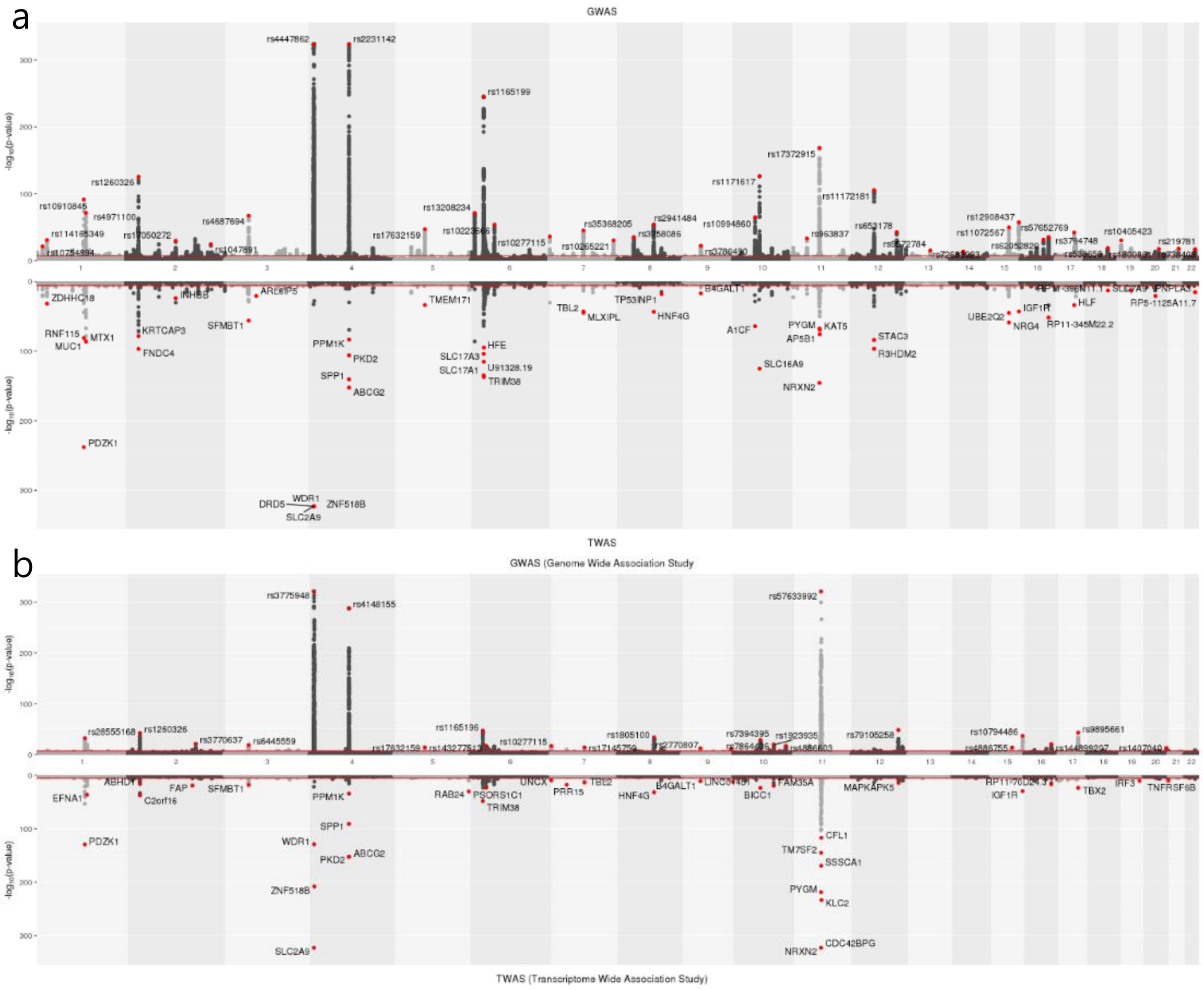

Supplementary Fig. 2 Comparison of effect sizes for the GWAS using UKBB-provided PCs and GWAS using newly calculated PCs in UKBB European. GWAS, genome-wide association study; PC, principal component; UKBB, UK BioBank.

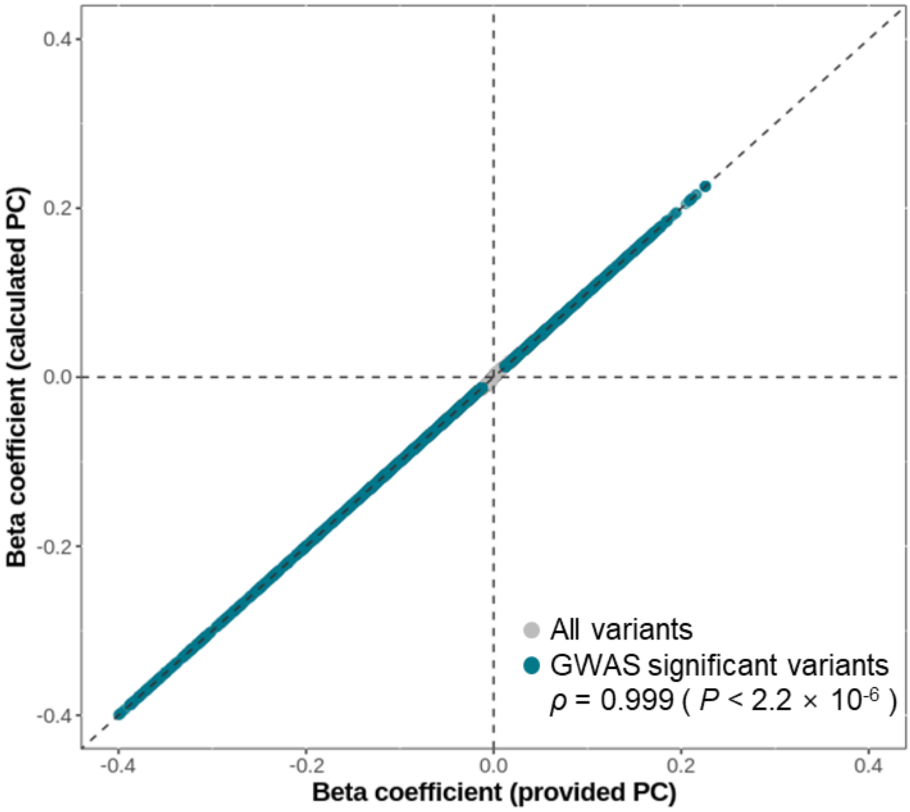

Supplementary Fig. 3. Comparison of effect sizes for the GWAS using UKBB-provided PCs and the GWAS using newly calculated PCs in UKBB non-European

(a) UKBB non-European PCA plot (UKBB-provided PC). (b) Comparison of effect sizes between the two GWASs. GWAS, genome-wide association study; PC, principal component; UKBB, UK BioBank.

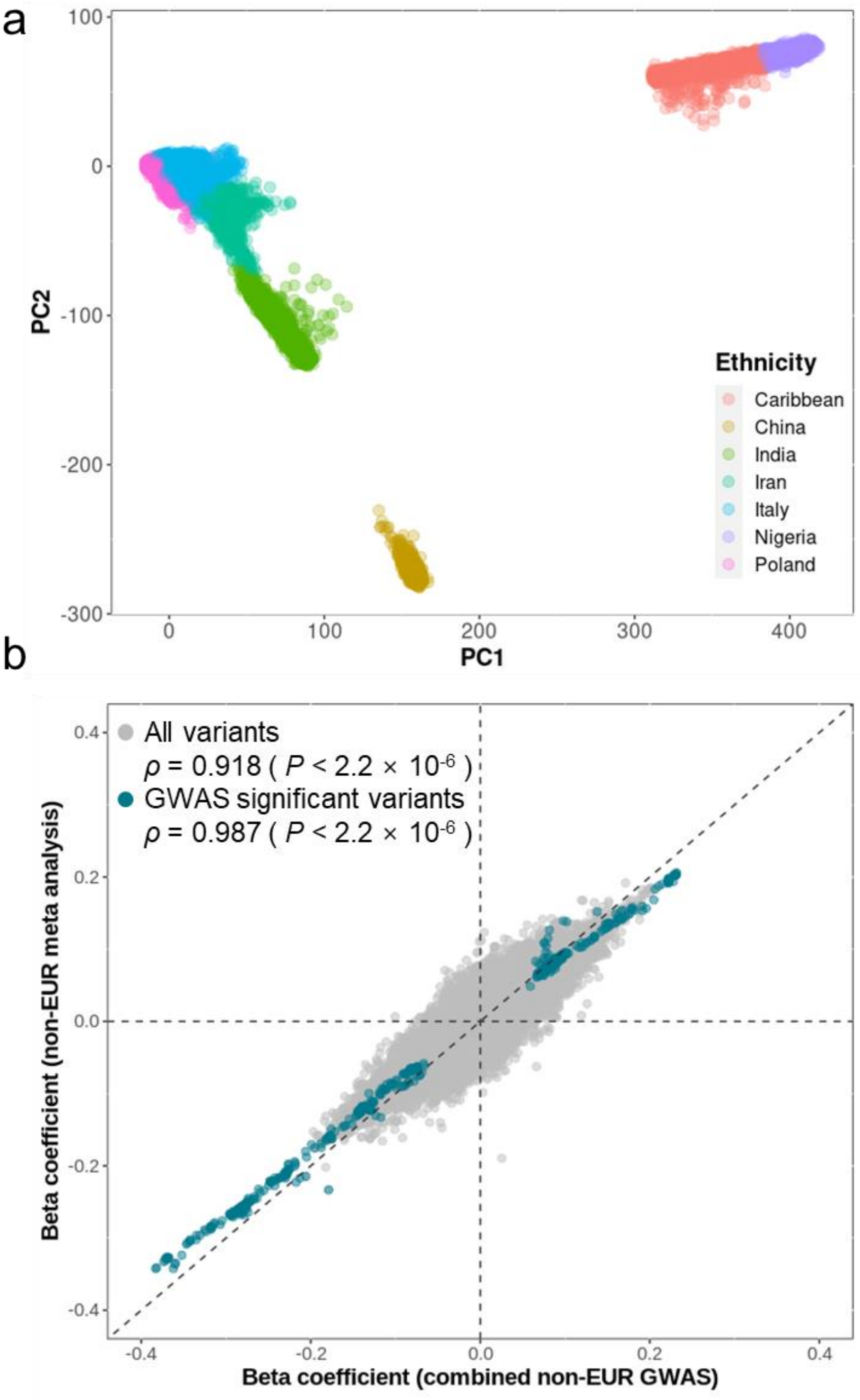

Supplementary Fig. 4 Comparison of the effect size and direction of the lead variants in the cross-ancestry meta-analysis with those in the GWAS of each UKBB non-European population

GWAS, genome-wide association study; UKBB, UK BioBank.

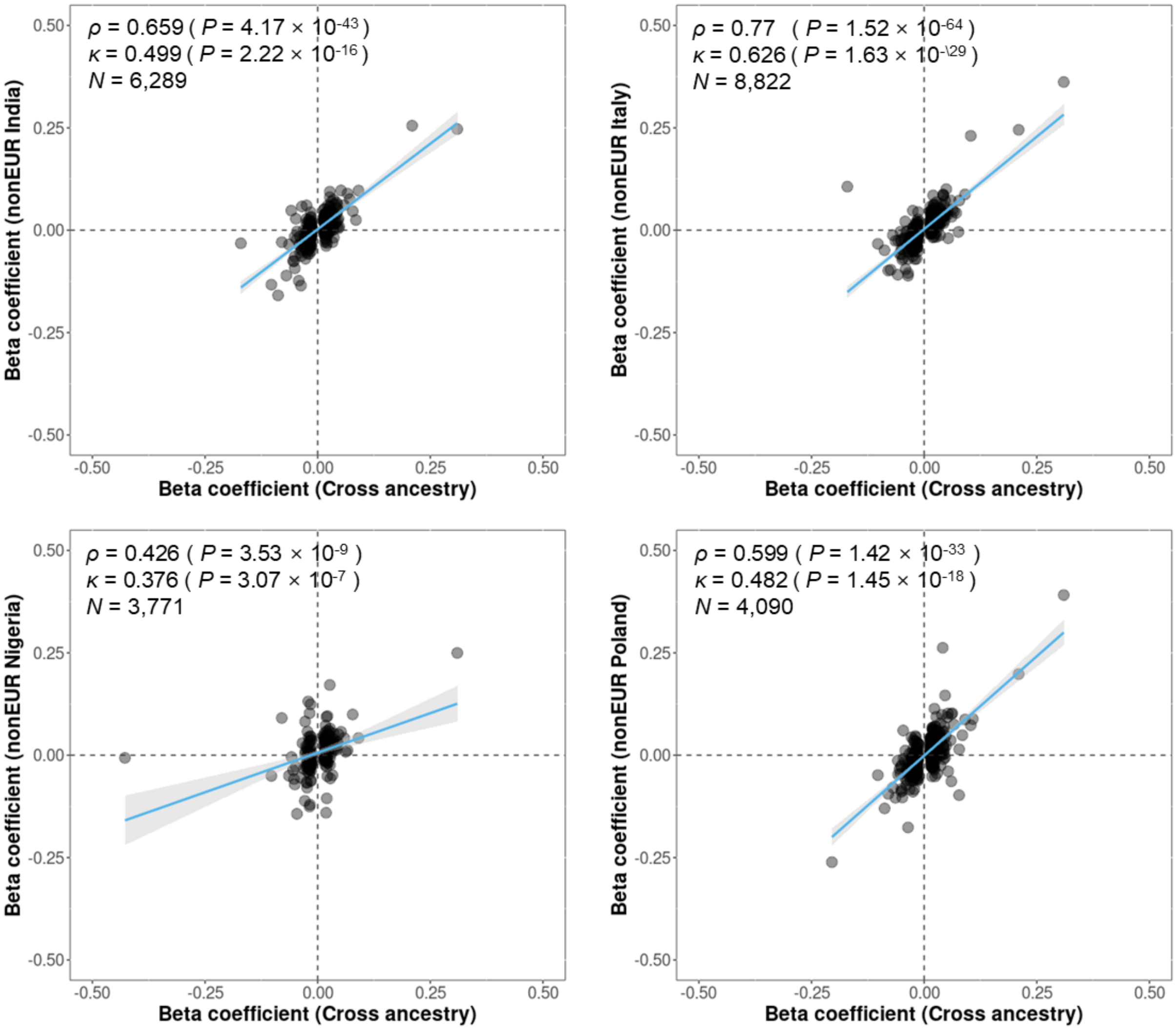

Supplementary Fig. 5 Regional plots of 17 previously unreported significant loci in the cross-ancestral meta-analysis

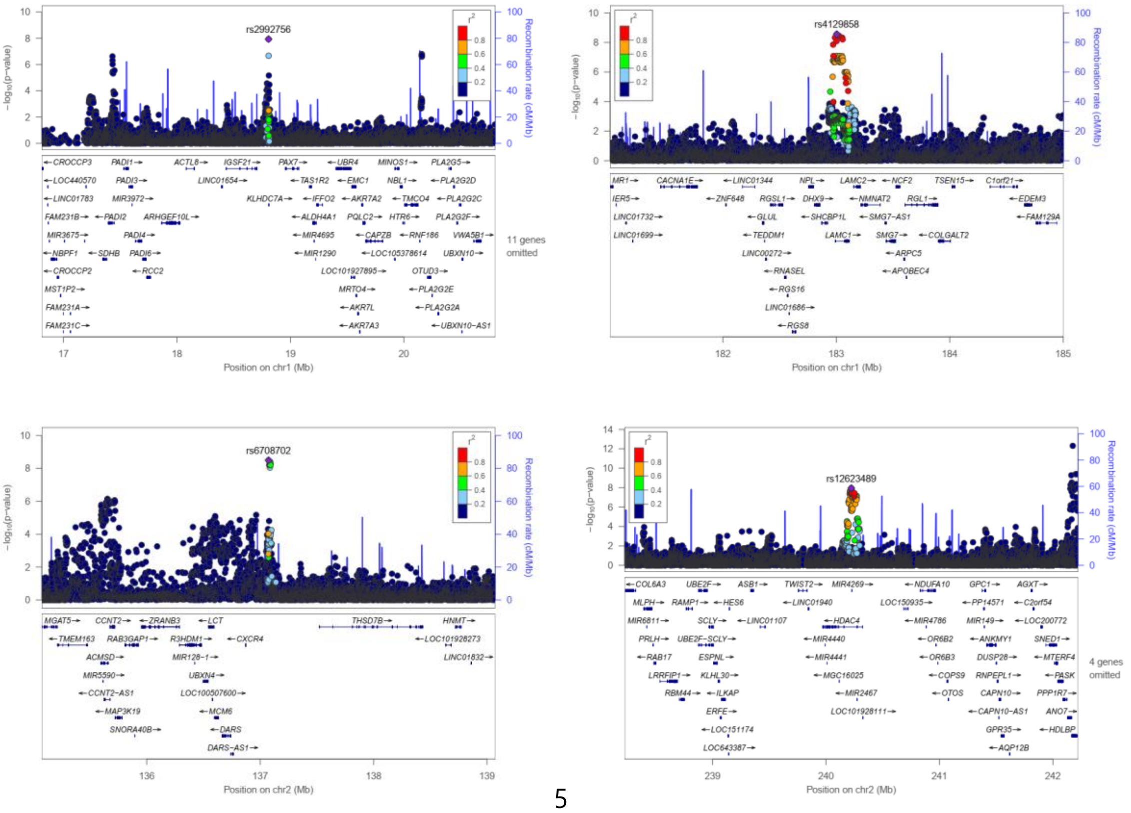

5

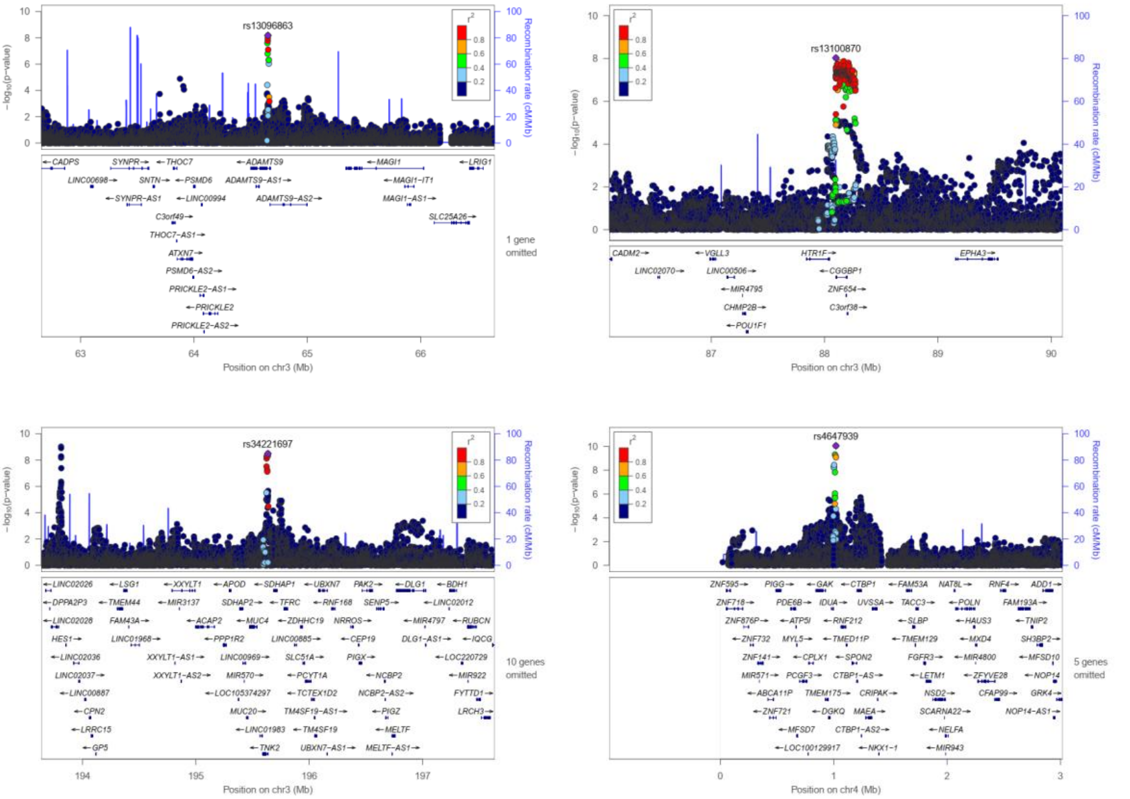

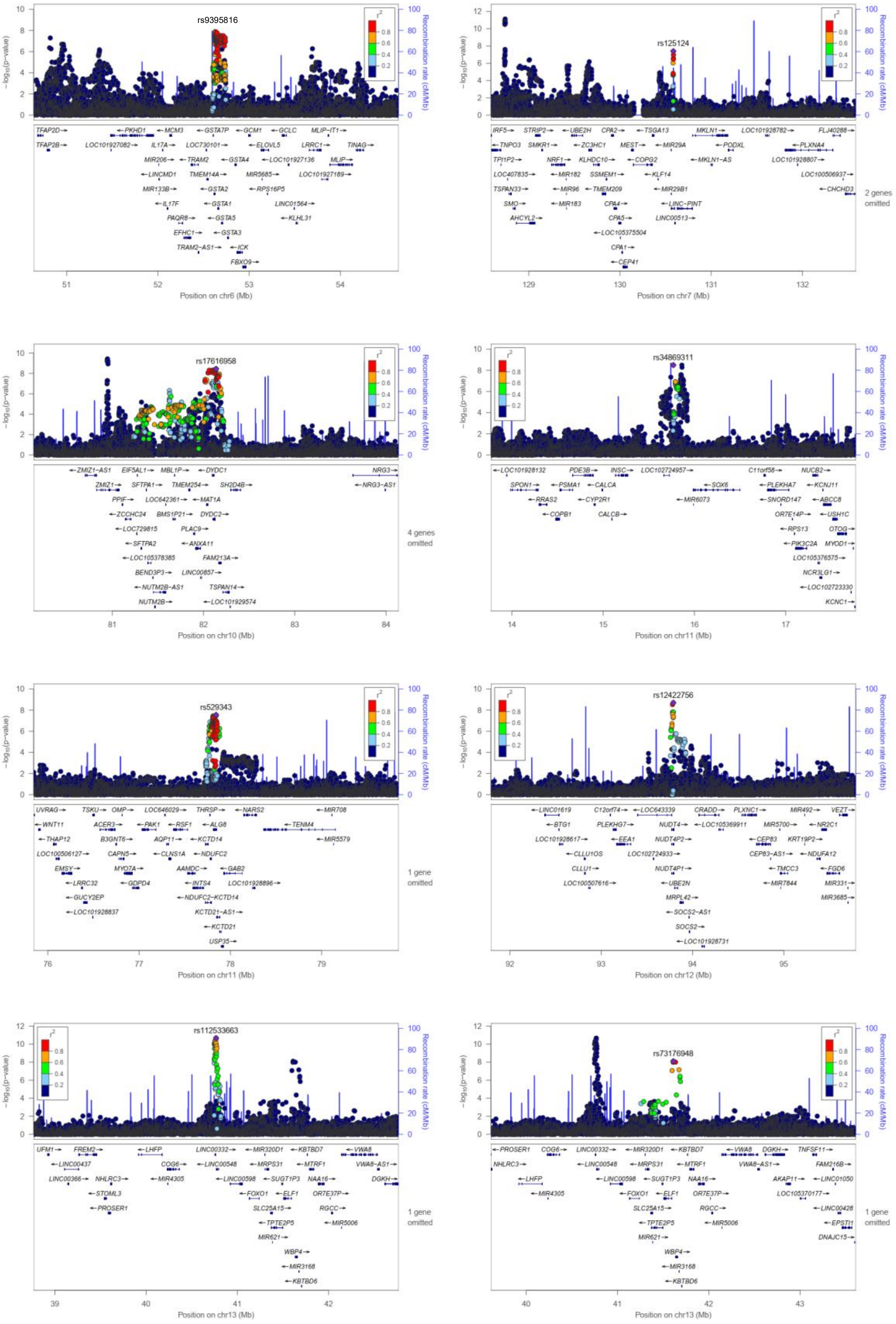
rs9395816
Position on chr6 (Mb)
rs125124
Position on chr7 (Mb)
rs17616958
Position on chr10 (Mb)
rs34869311
Position on chr11 (Mb)
rs529343
Position on chr11 (Mb)
rs12422756
Position on chr12 (Mb)
rs112533663
Position on chr13 (Mb)
rs73176948
Position on chr13 (Mb)
−log10(p−value)
Recombination rate (cM/Mb)

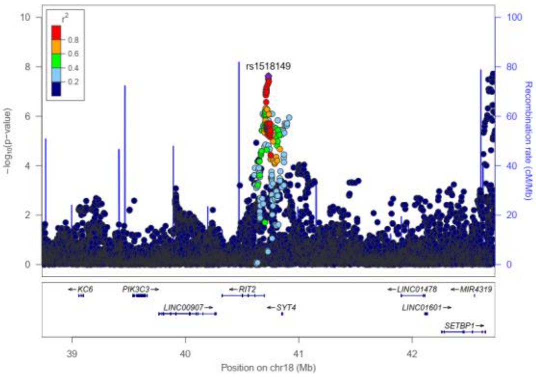

r2
0.8
0.6
0.4
0.2
rs1518149
-log10(p-value)
Recombination rate (cM/Mb)
KC6
PIK3C3
LINC00907
RIT2
SYT4
LINC01478
LINC01601
MIR4319
SETBP1
Position on chr18 (Mb)

Supplementary Fig. 6 Genetic correlations between traits according to ancestry

(a) Genetic correlations of the European ancestry. (b) Genetic correlations of the East Asian ancestry.

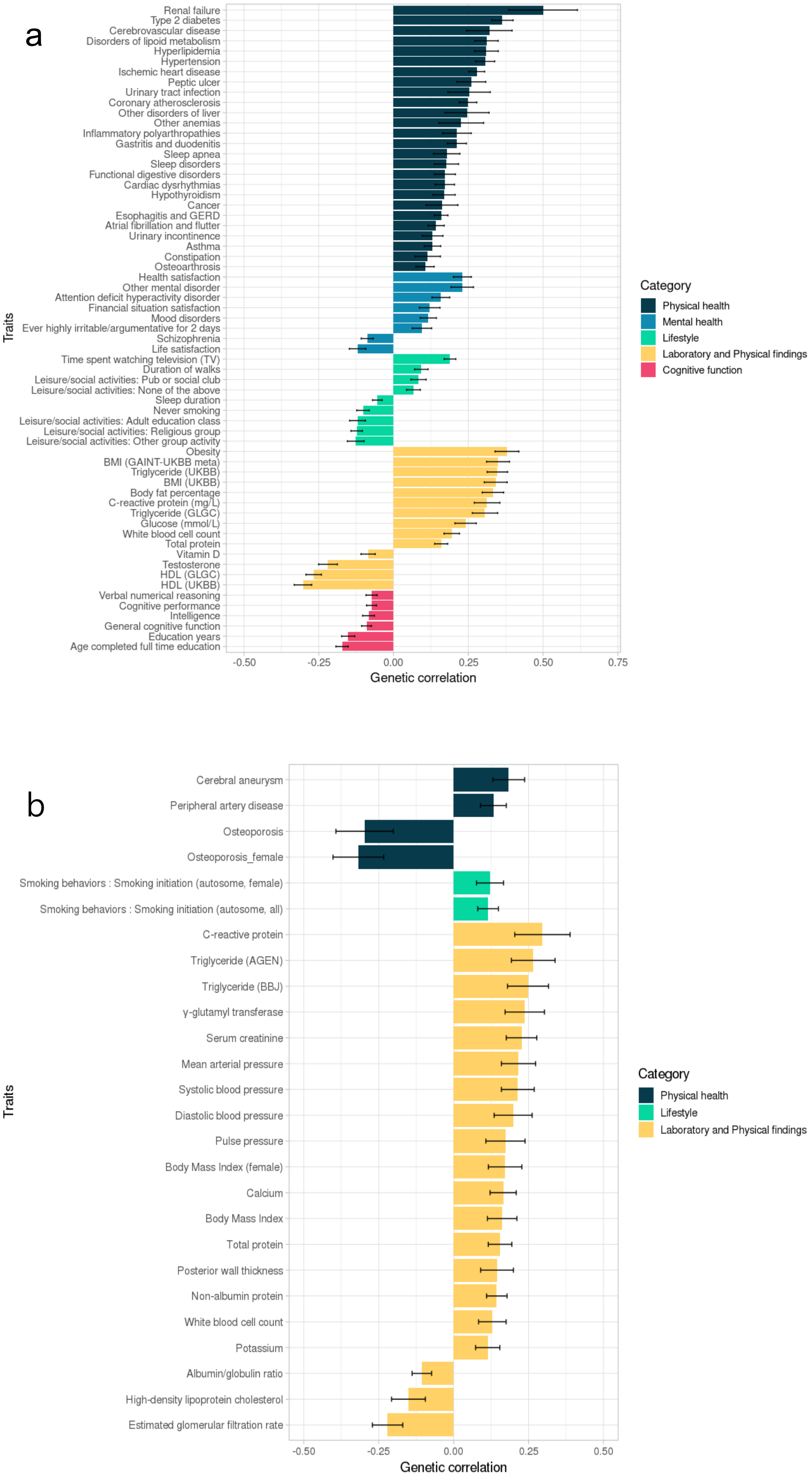

Supplementary Fig. 7 Functional enrichment analysis in the European and East Asian ancestry meta-analyses

(a) Tissue enrichment in individuals of European ancestry. (b) Tissue enrichment in East Asian ancestry.

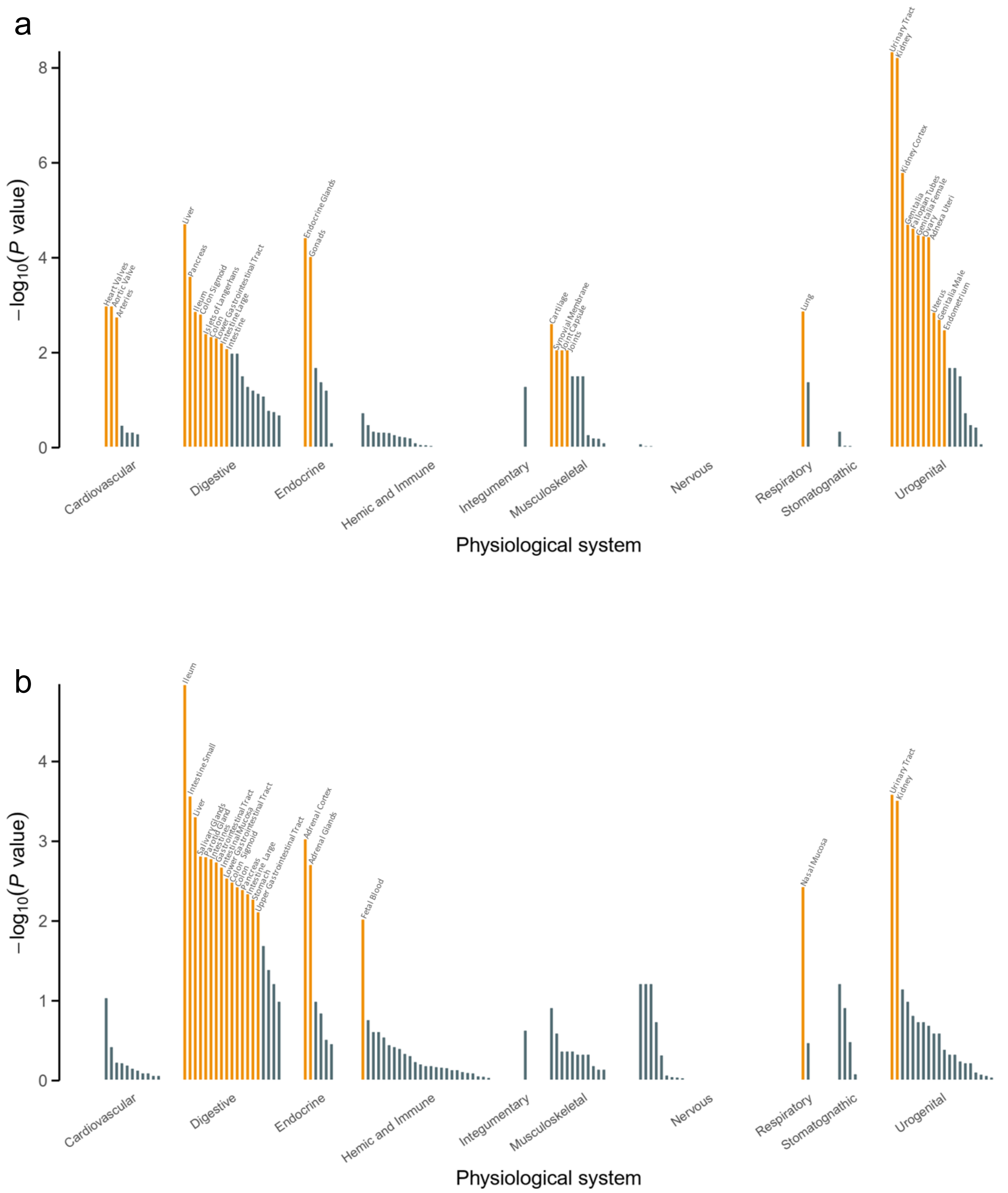

Supplementary Fig. 8 Leave-one-out polygenic risk score (LOO PRS)

(a) LOO PRS of European ancestry. (b) LOO PRS for East Asian ancestry. GWAS, genome-wide association study; UKBB, UK BioBank; KoGES, Korean Genome and Epidemiology study; CKDgen, Chronic Kidney Disease Genetics Consortium; BBJ, Biobank Japan; PRS, polygenic risk score.

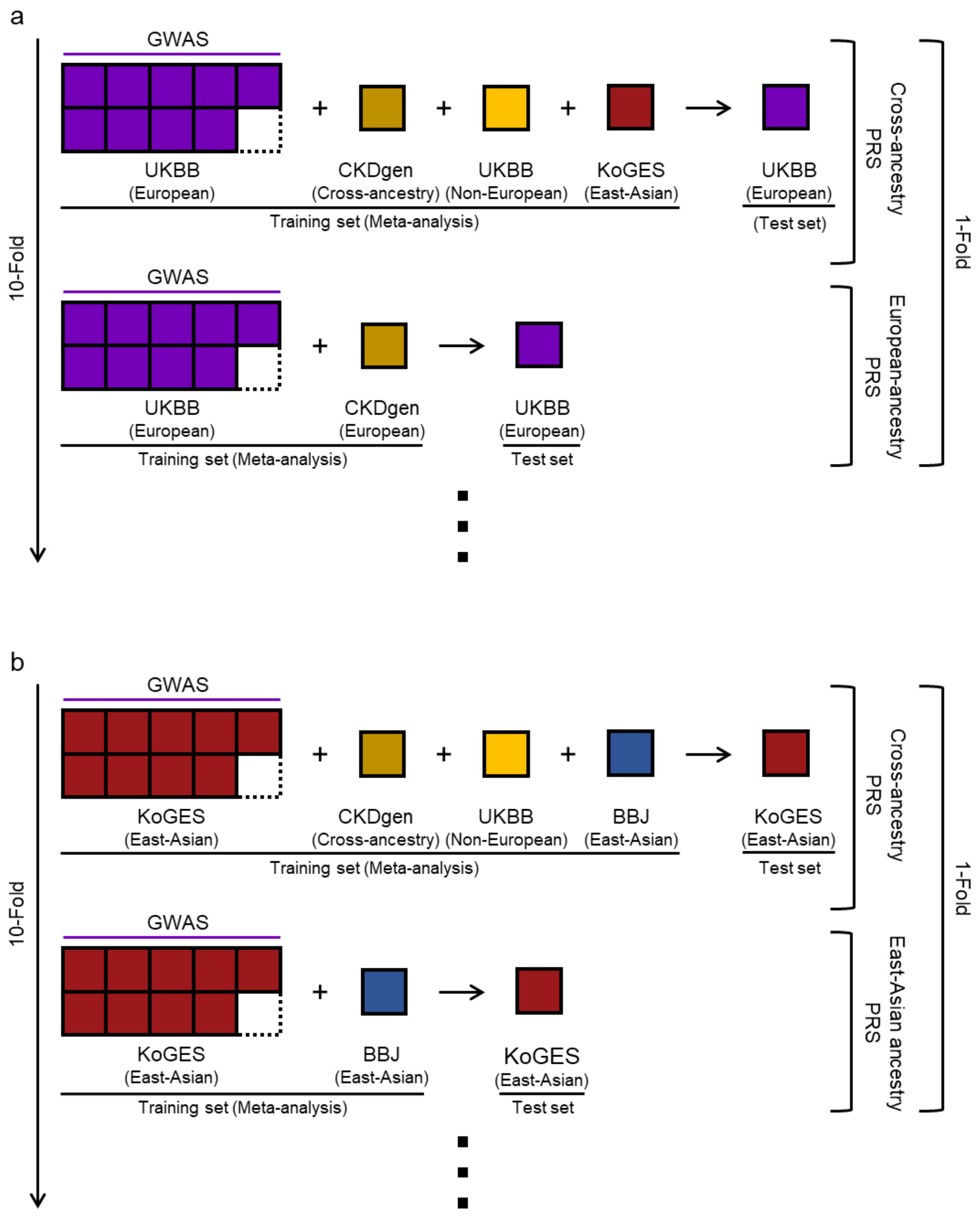

Supplementary Fig. 9 PRS phenome-wide association study (PheWAS) plot applying the PRS of European ancestry to the European population (UKBB)

UKBB, UK BioBank; PRS, polygenic risk score.

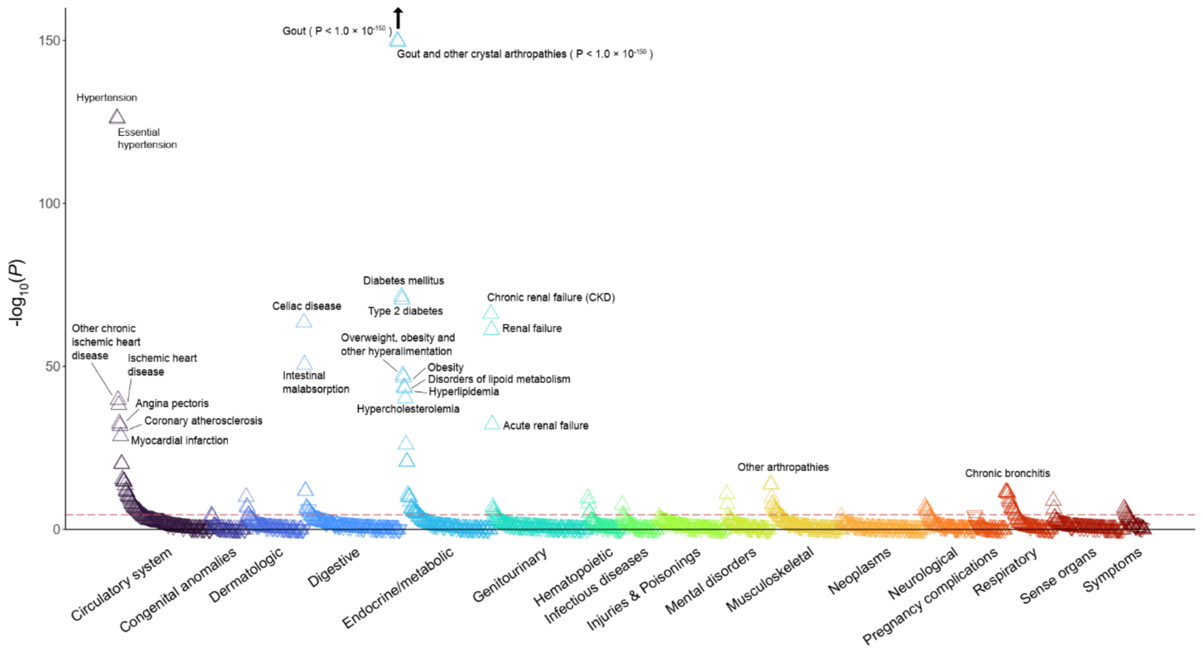

Supplementary Fig. 10 Kaplan-Meier survival curves for gout, heart failure, and essential hypertension with the cross-ancestry and European ancestry PRS

PRS, polygenic risk score.

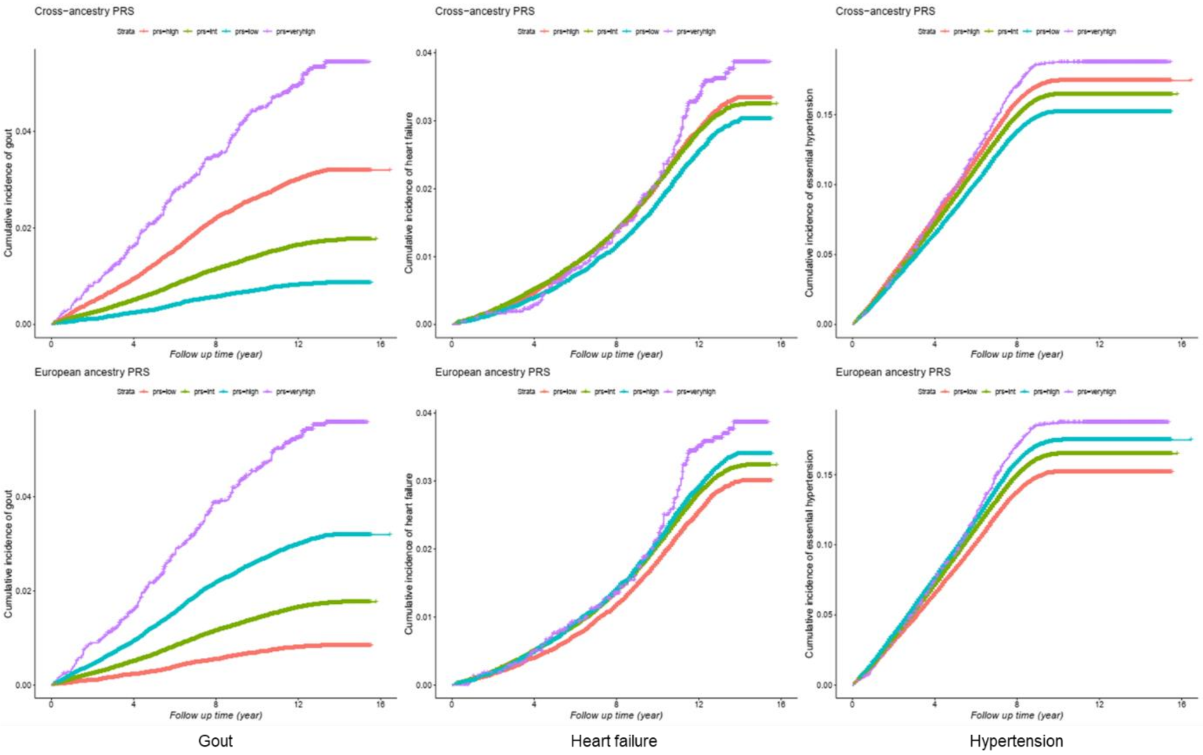

Supplementary Fig. 11 Comparison of disease prevalence according to PRS ancestry in the Korean population

(a) Hypertension (b) Gout. PRS, polygenic risk score.

a

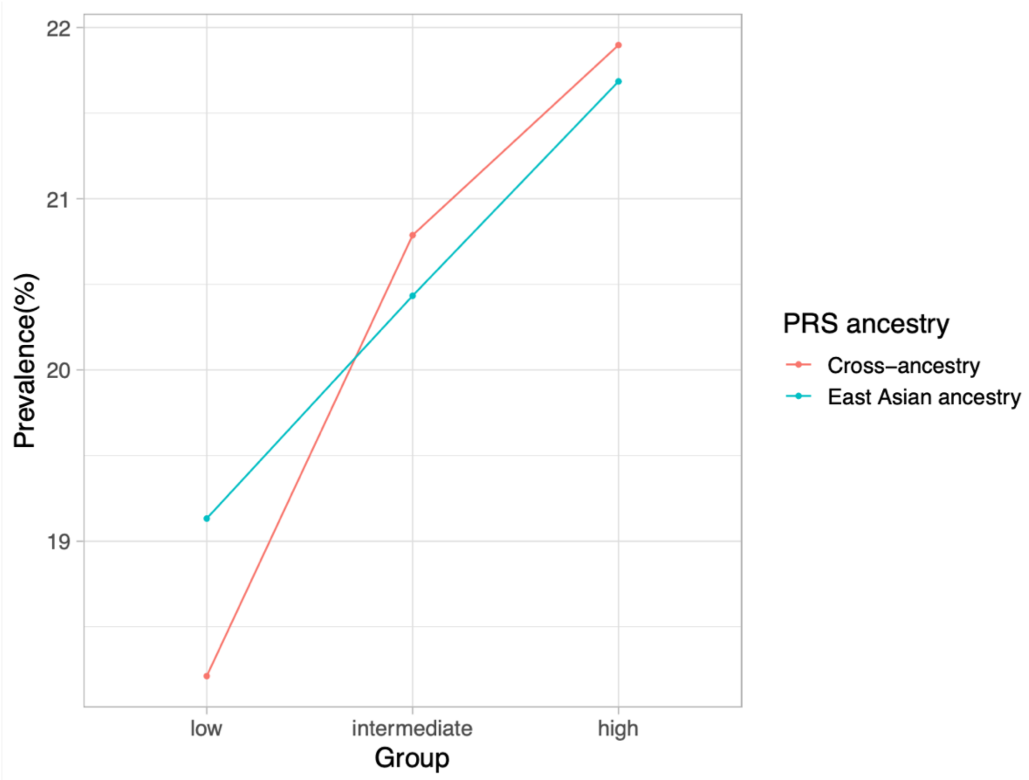

b

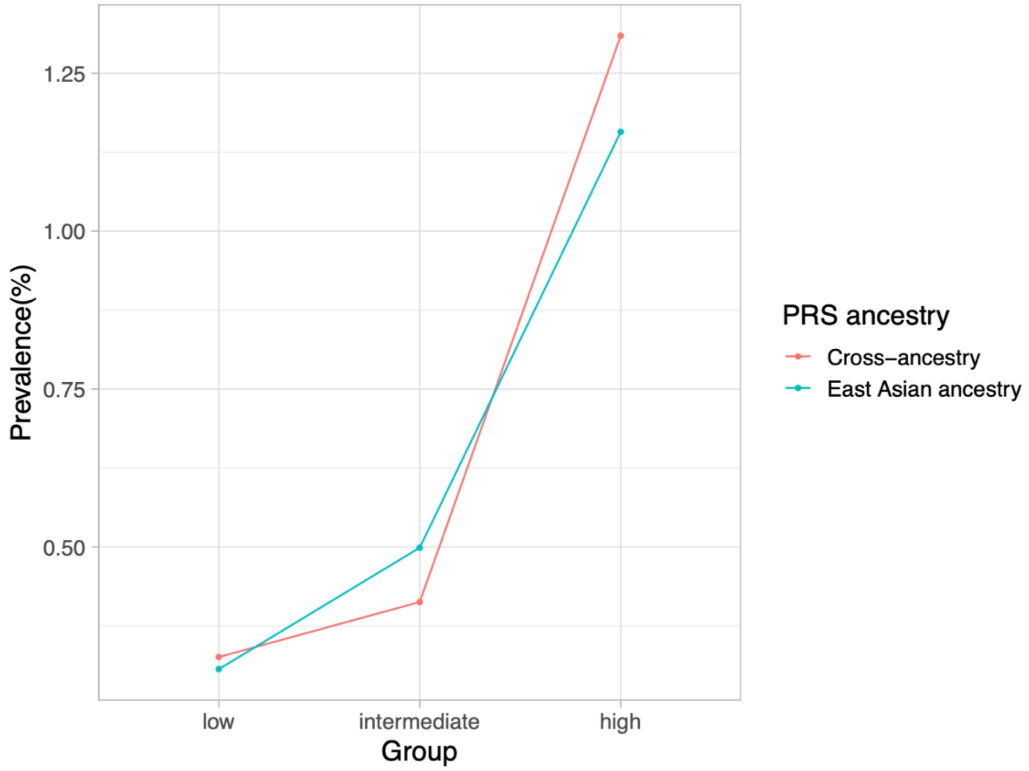

Supplementary Fig. 12. Comparison of odds ratios by PRS ancestry in the Korean population (a) Hypertension (b) Gout. PRS, polygenic risk score.

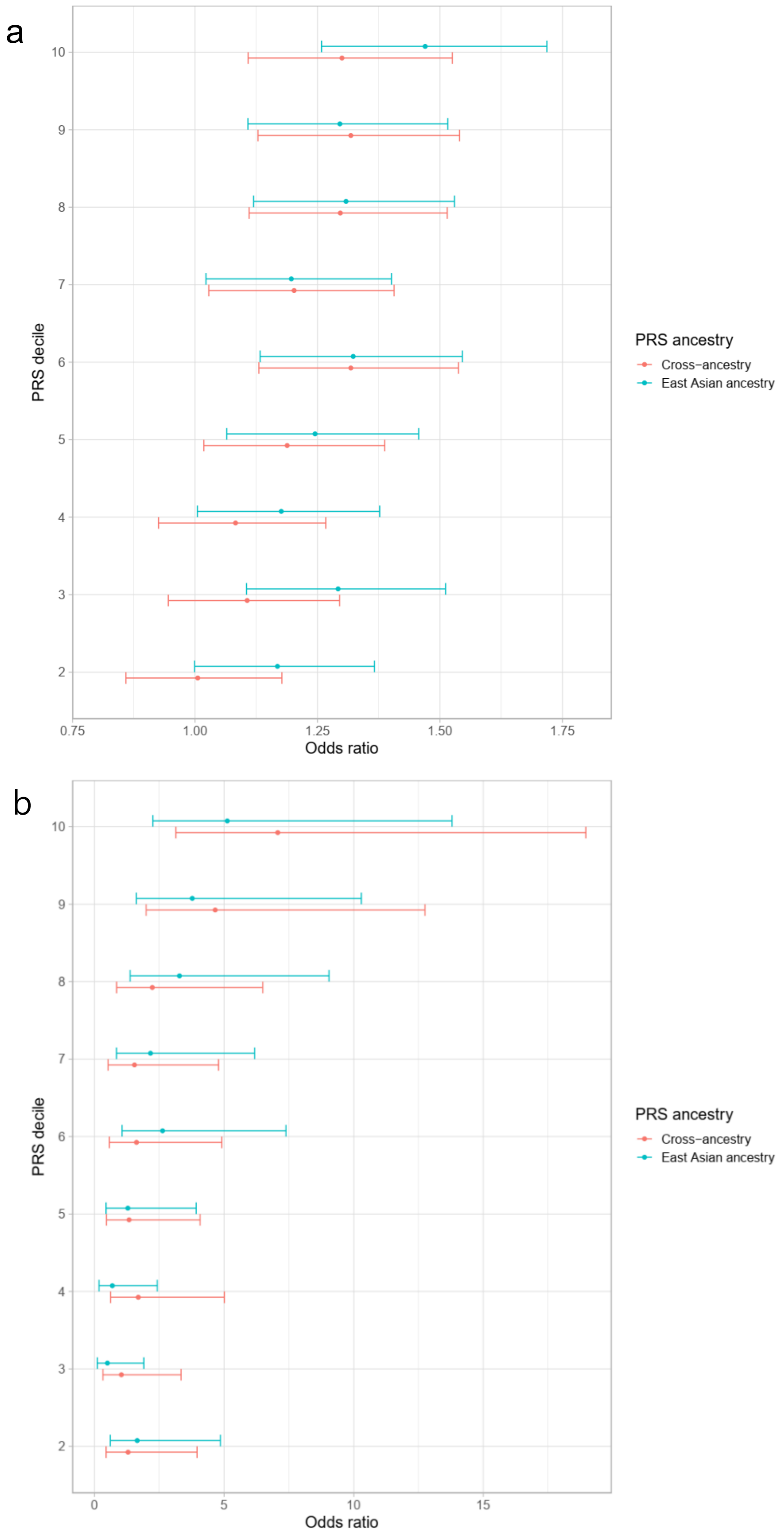

Supplementary Fig. 13 . Comparison of ROC curves of the PRS of the East Asian ancestry in the Korean population

(a) Hypertension (b) Gout. ROC, receiver operating characteristic; PRS, polygenic risk score; AUC, area under the ROC curve.

a

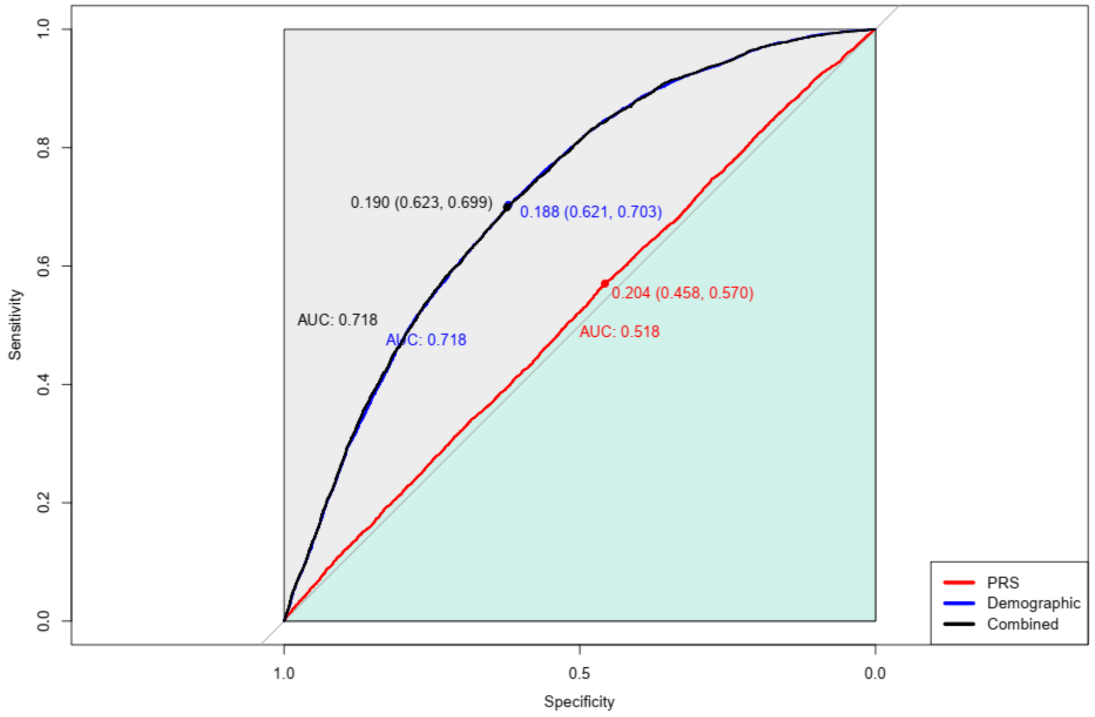

b

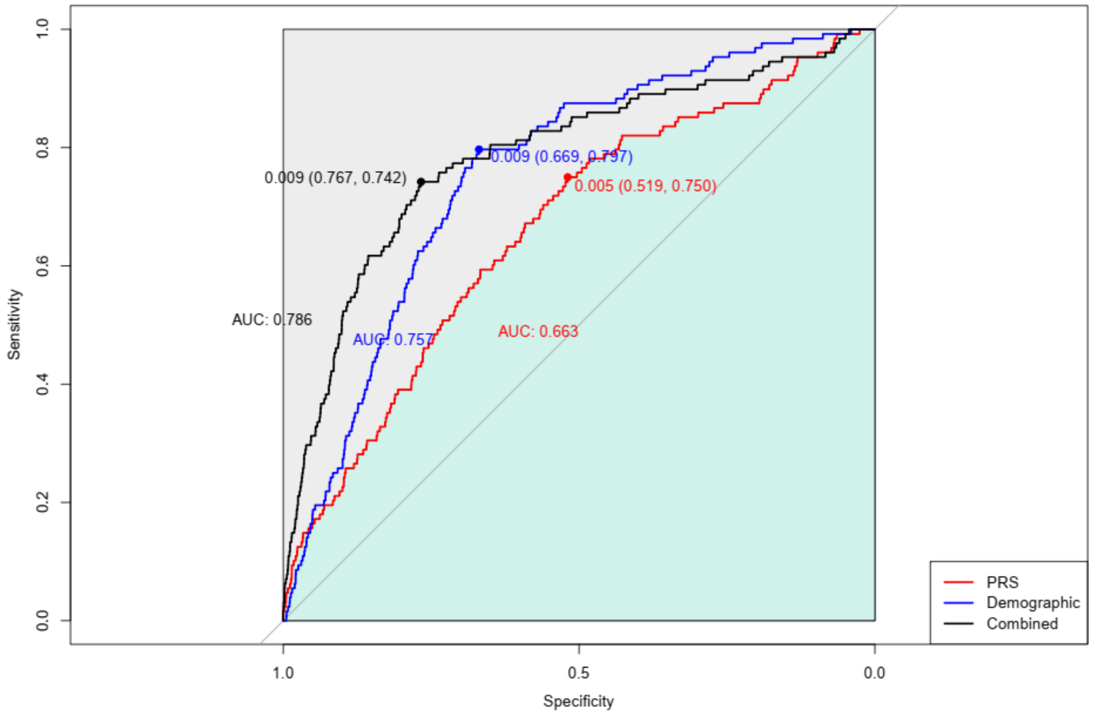

Supplementary Fig. 14 Comparison of ROC curves by cross-ancestry PRS in the Korean population

(a) Hypertension (b) Gout. ROC, receiver operating characteristic; PRS, polygenic risk score; AUC, area under the ROC curve

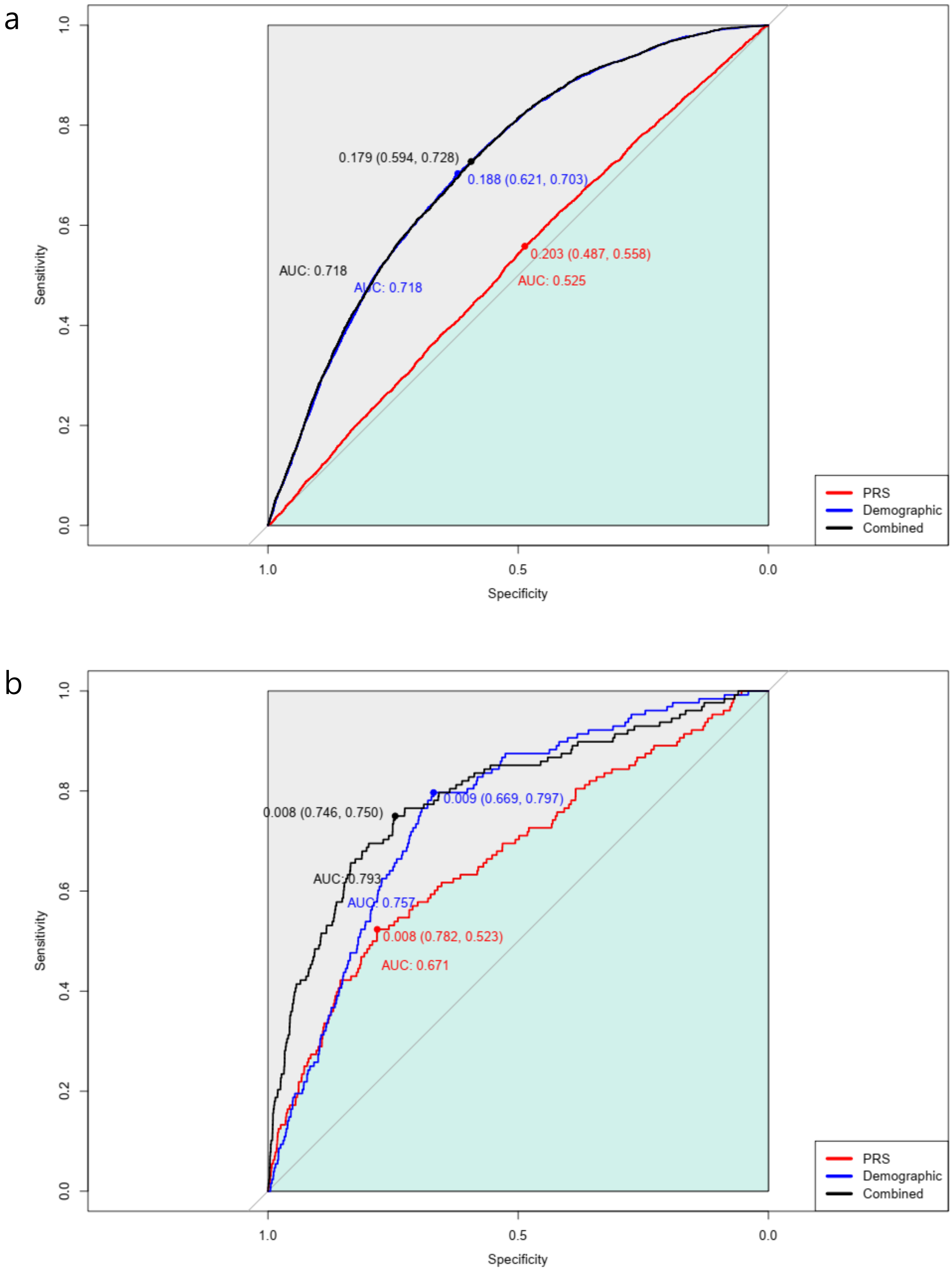

URLs

BOLT-LMM v2.3.4, https://alkesgroup.broadinstitute.org/BOLT-LMM/BOLT-LMM_manual.html;
KING v2.1, https://www.kingrelatedness.com;
Eagle v2.3, https://alkesgroup.broadinstitute.org/Eagle;
IMPUTE v4, https://jmarchini.org/software/#impute-4;
EPACTS package v3.2.6, https://genome.sph.umich.edu/wiki/EPACTS;
GWAtoolbox v2.2.4-10, https://rdrr.io/rforge/GWAtoolbox;
METAL (released on 2011-03-25), https://genome.sph.umich.edu/wiki/METAL;
ANNOVAR (released 2019-09-27), https://annovar.openbioinformatics.org/en/latest/user-guide/download;
LocusZoom v1.4, http://locuszoom.org;
LDSC v1.0.1, https://github.com/bulik/Idsc;
DEPICT v1.1, https://github.com/perslab/depict;
GSA-SNP2 (released 2020-09-01), https://sites.google.com/view/gsasnp2;
coloc v5.1.1, https://chrlswallace.github.io/coloc/articles/a01_intro.html;
PrediXcan v0.7.5, https://github.com/hakyimlab/PrediXcan;
PRS-CS (released on 2021-06-04), https://github.com/getian107/PRScs;
pROC v1.18.0, https://www.rdocumentation.org/packages/pROC;
survplot, https://www.rdocumentation.org/packages/rms/versions/6.7-0/topics/survplot;
TwoSampleMR v0.5.6, https://mrcieu.github.iofTwoSampleMR/news/index.html#twosamplemr-v056;
MR-PRESSO v1.0, https://github.com/rondolab/MR-PRESSO;
SMR v1.3.1, https://yanglab.westlake.edu.cn/software/smr;
SAIGE v1.1.3,https://github.com/weizhouUMICH/SAIGE;
PLINK v1.9, https://www.cog-genomics.org/plink/1.9/;
PLINK v2.0, https://www.cog-genomics.org/plink/2.0/.
