# Supplementary material for: Large-scale cross-ancestry genome-wide meta-analysis of serum urate
Source: Nat Commun. 2024 Apr 24;15:3441. doi: 10.1038/s41467-024-47805-4 (PMC11043400; doi:10.1038/s41467-024-47805-4)
Supplement: Supplementary file 3 — Description of Additional Supplementary Files [file 41467_2024_47805_MOESM3_ESM.pdf]

Description of Additional Supplementary Files

File Name: Supplementary Data 1
Description: Information on cohorts used in meta-analysis.
Abbreviations: CKDgen, Chronic Kidney Disease Genetics Consortium; UKBB, UK Biobank; BBJ, Biobank Japan; KoGES, Korean Genome and Epidemiology Study; SU, serum urate; IQ, imputation quality; MAF, minor allele frequency.

File Name: Supplementary Data 2
Description: Lead loci of cross-ancestry meta-analysis. P-values were determined using a two-sided test. Lead loci that passed the Bonferroni correction were considered significant (P ≤ 5E-08).
Abbreviations: SNP, single nucleotide polymorphism; UTR3, three prime untranslated region; ncRNA, non-coding RNA.

File Name: Supplementary Data 3
Description: Lead loci of European ancestry meta-analysis. P-values were determined using a two-sided test. Lead loci that passed the Bonferroni correction were considered significant (P ≤ 5E-08).
Abbreviations: SNP, single nucleotide polymorphism; UTR3, three prime untranslated region; ncRNA, non-coding RNA.

File Name: Supplementary Data 4
Description: Lead loci of East Asian ancestry meta-analysis. P-values were determined using a two-sided test. Lead loci that passed the Bonferroni correction were considered significant (P ≤ 5E-08).
Abbreviations: SNP, single nucleotide polymorphism; UTR3, three prime untranslated region; ncRNA, non-coding RNA.

File Name: Supplementary Data 5
Description: Genetic correlation between European and East Asian ancestry. P-value reported is for a test that the heritability is greater than 0: P(h^2 > 0.0).
Abbreviations: Val (Obs), common-SNP observed scale; SE, standard error; Z, Z-score; h, heritability; pgi, genetic impact correlation; pge, genetic effect correlation.

File Name: Supplementary Data 6
Description: Set-specific lead loci by ancestry. P-values were determined using a two-sided test. Lead loci that passed the Bonferroni correction were considered significant (P ≤ 5E-08).
Abbreviations: SNP, single nucleotide polymorphism; UTR3, three prime untranslated region; ncRNA, non-coding RNA.

File Name: Supplementary Data 7
Description: Genetic heritability of European and East Asian ancestry.
Abbreviations: SNP, single nucleotide polymorphism; h, heritability.

File Name: Supplementary Data 8
Description: Significant results of tissue enrichment analysis (FDR < 0.05). P-values were determined using a two-sided test. Significant results that passed the FDR correction (FDR P ≤ 0.05).
Abbreviations: MeSH, medical subject headings.

File Name: Supplementary Data 9
Description: Significant results of gene set enrichment analysis (FDR < 0.25). P-values were determined using a two-sided test. Q-values is FDR adjusted P-value. Significant results that passed the FDR correction (FDR ≤ 0.05).
Abbreviations: KEGG, Kyoto Encyclopedia of Genes and Genomes; NABA, matrisome Project; REACTOME, Reactome Project.

File Name: Supplementary Data 10
Description: Colocalized results in the cross-ancestry meta-analysis (PP.H4 > 0.8). Each H represents different posterior probabilities as follows: H0 indicates no causal variant; H1 denotes a causal variant for trait 1 only; H2 indicates causal variant for trait only 2; H3 suggests two distinct causal variants; and H4 points to one common causal variant.
Abbreviations: PP, posterior probability; minbp, minimum base pair; maxbp, maximum base pair, eqtl, expression qunatitative trait loci.

File Name: Supplementary Data 11
Description: Colocalized results in the European meta-analysis (PP.H4 > 0.8). Each H represents different posterior probabilities as follows: H0 indicates no causal variant; H1 denotes a causal variant for trait 1 only; H2 indicates causal variant for trait only 2; H3 suggests two distinct causal variants; and H4 points to one common causal variant.
Abbreviations: PP, posterior probability; minbp, minimum base pair; maxbp, maximum base pair, eqtl, expression qunatitative trait loci.

File Name: Supplementary Data 12
Description: Colocalized results in the East Asian meta-analysis (PP.H4 > 0.8). Each H represents different posterior probabilities as follows: H0 indicates no causal variant; H1 denotes a causal variant for trait 1 only; H2 indicates causal variant for trait only 2; H3 suggests two distinct causal variants; and H4 points to one common causal variant.
Abbreviations: PP, posterior probability; minbp, minimum base pair; maxbp, maximum base pair, eqtl, expression qunatitative trait loci.

File Name: Supplementary Data 13
Description: Significant results of the transcriptome-wide association study in the cross-ancestry meta-analysis (Bonferroni correction). P-values were determined using a two-sided test. Significant results that passed the Bonferroni correction.
Abbreviations: CHR, chromosome.

File Name: Supplementary Data 14
Description: Significant results of the transcriptome-wide association study in the European ancestry meta-analysis (Bonferroni correction). P-values were determined using a two-sided test. Significant results that passed the Bonferroni correction.
Abbreviations: CHR, chromosome.

File Name: Supplementary Data 15
Description: Significant results of the transcriptome-wide association study in the East Asian ancestry meta-analysis (Bonferroni correction). P-values were determined using a two-sided test. Significant results that passed the Bonferroni correction.
Abbreviations: CHR, chromosome.

File Name: Supplementary Data 16
Description: Significant results of the PRS phenome-wide association study using cross-ancestry PRS in the European population (Bonferroni correction). The P-value of PRS corrected for age and sex. P-values were determined using a two-sided test. Significant results that passed the Bonferroni correction. The R^2 is the Nagelkerke pseudo R^2 of PRS.
Abbreviations: R, coefficient of determination; PRS, polygenic risk score.

File Name: Supplementary Data 17
Description: Significant results of the PRS phenome-wide association study using European ancestry PRS in the European population (Bonferroni correction). The P-value of PRS corrected for age and sex. P-values were determined using a two-sided test. Significant results that passed the Bonferroni correction. The R^2 is the Nagelkerke pseudo R^2 of PRS.
Abbreviations: R, coefficient of determination; PRS, polygenic risk score.

File Name: Supplementary Data 18
Description: Significant results of the PRS phenome-wide association study using European ancestry PRS in the European population (Bonferroni correction). The P-value of PRS corrected for age and sex. P-values were determined using a two-sided test. Significant results that passed the Bonferroni correction. The R^2 is the Nagelkerke pseudo R^2 of PRS.
Abbreviations: R, coefficient of determination; OR, odds ratio; PRS, polygenic risk score.

File Name: Supplementary Data 19
Description: Baseline characteristics of the study population compared by SU cross-ancestry PRS group. P-values were determined using a two-sided test.
Abbreviations: SU, serum urate; C.I., confidence interval; PRS, polygenic risk score.

File Name: Supplementary Data 20
Description: Baseline characteristics of the study population compared by SU European ancestry PRS group. P-values were determined using a two-sided test.
Abbreviations: SU, serum urate; C.I., confidence interval; PRS, polygenic risk score.

File Name: Supplementary Data 21
Description: Hazard ratios and 95% confidence intervals of gout, heart failure, and essential hypertension by cross-ancestry PRS and European ancestry PRS without ULT.
Abbreviations: SU, serum urate; C.I., confidence interval; PRS, polygenic risk score; HR, hazard ratio.

File Name: Supplementary Data 22
Description: Disease prevalence by PRS ancestry in the Korean population for gout and hypertension.
Abbreviations: SU, serum urate; PRS, polygenic risk score.

File Name: Supplementary Data 23
Description: Odds ratios according to PRS by ancestry in the Korean population for gout and hypertension. P-values were determined using a two-sided test.
Abbreviations: SU, serum urate; C.I., confidence interval; PRS, polygenic risk score.

File Name: Supplementary Data 24
Description: ROC curves by PRS ancestry in the Korean population for gout and hypertension.
Abbreviations: SU, serum urate; PRS, polygenic risk score.

File Name: Supplementary Data 25
Description Two-Sample Mendelian randomization analysis results between SU and gout, heart failure, and essential hypertension. P-values were determined using a two-sided test.
Abbreviations: SU, serum urate; SE, standard error; MR, mendelian randomization.

File Name: Supplementary Data 26
Description: MR association between SU-related gene expression and SU. P-values were determined using a two-sided test.
Abbreviations: SU, serum urate; MR, mendelian randomization; eQTL, expression qunatitative trait loci; SNP, single nucleotide polymorphism; CHR, chromosome; SE, standard error; HEIDI, heterogeneity in dependent instruments.

File Name: Supplementary Data 27
Description: MR association between SU-related gene expression and gout, heart failure, and essential hypertension. P-values were determined using a two-sided test. Significant results that passed the FDR correction (FDR $\leq$ 0.05).
Abbreviations: SU, serum urate; MR, mendelian randomization; eQTL, expression qunatitative trait loci; SNP, single nucleotide polymorphism; CHR, chromosome; SE, standard error.

File Name: Supplementary Data 28
Description: MR mediation results on gout, heart failure, and essential hypertension via SU. P-values were determined using a two-sided test.
Abbreviations: SU, serum urate; MR, mendelian randomization; eQTL, expression qunatitative trait loci; SE, standard error.
