# Supplementary material for: Large-scale cross-ancestry genome-wide meta-analysis of serum urate
Source: Nat Commun. 2024 Apr 24;15:3441. doi: 10.1038/s41467-024-47805-4 (PMC11043400; doi:10.1038/s41467-024-47805-4)
Supplement: Supplementary file 5 — Reporting Summary [file 41467_2024_47805_MOESM5_ESM.pdf]

nature portfolio

Corresponding author(s): Young Jin Kim
Hong-Hee Won

Last updated by author(s): Mar 15, 2024

Reporting Summary

Nature Portfolio wishes to improve the reproducibility of the work that we publish. This form provides structure for consistency and transparency in reporting. For further information on Nature Portfolio policies, see our Editorial Policies and the Editorial Policy Checklist.

Statistics

For all statistical analyses, confirm that the following items are present in the figure legend, table legend, main text, or Methods section.

n/a	Confirmed	
☐	☒	The exact sample size (*n*) for each experimental group/condition, given as a discrete number and unit of measurement
☐	☒	A statement on whether measurements were taken from distinct samples or whether the same sample was measured repeatedly
☐	☒	The statistical test(s) used AND whether they are one- or two-sided<br>*Only common tests should be described solely by name; describe more complex techniques in the Methods section.*
☐	☒	A description of all covariates tested
☐	☒	A description of any assumptions or corrections, such as tests of normality and adjustment for multiple comparisons
☐	☒	A full description of the statistical parameters including central tendency (e.g. means) or other basic estimates (e.g. regression coefficient) AND variation (e.g. standard deviation) or associated estimates of uncertainty (e.g. confidence intervals)
☐	☒	For null hypothesis testing, the test statistic (e.g. *F*, *t*, *r*) with confidence intervals, effect sizes, degrees of freedom and *P* value noted<br>*Give P values as exact values whenever suitable.*
☒	☐	For Bayesian analysis, information on the choice of priors and Markov chain Monte Carlo settings
☒	☐	For hierarchical and complex designs, identification of the appropriate level for tests and full reporting of outcomes
☐	☒	Estimates of effect sizes (e.g. Cohen's *d*, Pearson's *r*), indicating how they were calculated

Our web collection on statistics for biologists contains articles on many of the points above.

Software and code

Policy information about availability of computer code

Data collection	In this study, we performed a meta-analysis using six summary statistics for the four cohorts. The UK Biobank (UKBB) genotype and phenotype data are available by requesting access on the UKBB homepage (https://www.ukbiobank.ac.uk/). Summary statistics are publicly available from the Chronic Kidney Disease Genetics Consortium (CKDGen, http://ckdgen.imbi.uni-freiburg.de/). BBJ summary statistics were downloaded from the Biobank Japan PheWeb (https://pheweb.jp/). The full summary statistics of the Korea Biobank Array (KBA) GWAS are available at the NHGRI-EBI GWAS Catalog (https://www.ebi.ac.uk/gwas/downloads) and the Korea National Institute of Health PheWeb (https://coda.nih.go.kr/usab/pheweb/intro.do). The population-based cohorts in the KoGES, including the Ansan and Ansung study, the Health EXAminee (HEXA) study and the Cardiovascular Disease Association Study (CAVAS), consist of community-dwellers and participants recruited from the national health examinee registry, men and women, aged ≥ 40 years at baseline.
Data analysis	BOLT-LMM v2.3.4, https://alkesgroup.broadinstitute.org/BOLT-LMM/BOLT-LMM_manual.html;<br>KING v2.1, https://www.kingrelatedness.com;<br>Eagle v2.3, https://alkesgroup.broadinstitute.org/Eagle;<br>IMPUTE v4, https://jmarchini.org/software/#impute-4;<br>EPACTS package v3.2.6, https://genome.sph.umich.edu/wiki/EPACTS;<br>GWAtoolbox v2.2.4-10, https://rdrr.io/rforge/GWAtoolbox;<br>METAL (released on 2011-03-25), https://genome.sph.umich.edu/wiki/METAL;<br>ANNOVAR (released 2019-09-27), https://annovar.openbioinformatics.org/en/latest/user-guide/download;<br>LocusZoom v1.4, http://locuszoom.org;<br>LDSC v1.0.1, https://github.com/bulik/ldsc;<br>DEPICT v1.1, https://github.com/perslab/depict;<br>GSA-SNP2 (released 2020-09-01), https://sites.google.com/view/gsasnp2;coloc, https://chrlswallace.github.io/coloc/articles/a01_intro.html;

coloc v5.1.1, https://chrlswallace.github.io/coloc/articles/a01_intro.html;
PrediXcan v0.7.5, https://github.com/hakyimlab/PrediXcan;
PRS-CS (released on 2021-06-04), https://github.com/getian107/PRScs;
pROC v1.18.0, https://www.rdocumentation.org/packages/pROC;
survplot, https://www.rdocumentation.org/packages/rms/versions/6.7-0/topics/survplot;
TwoSampleMR v0.5.6, https://mrcieu.github.iofTwoSampleMR/news/index.html#twosamplemr-v056;
MR-PRESSO v1.0, https://github.com/rondolab/MR-PRESSO;
SMR v1.3.1, https://yanglab.westlake.edu.cn/software/smr;
SAIGE v1.1.3, https://github.com/weizhouUMICH/SAIGE;
PLINK v1.9, https://www.cog-genomics.org/plink/;
PLINK v2.0, https://www.cog-genomics.org/plink/2.0/.

For manuscripts utilizing custom algorithms or software that are central to the research but not yet described in published literature, software must be made available to editors and reviewers. We strongly encourage code deposition in a community repository (e.g. GitHub). See the Nature Portfolio guidelines for submitting code & software for further information.

Data

Policy information about availability of data

All manuscripts must include a data availability statement. This statement should provide the following information, where applicable:

- Accession codes, unique identifiers, or web links for publicly available datasets
- A description of any restrictions on data availability
- For clinical datasets or third party data, please ensure that the statement adheres to our policy

The full summary statistics of cross-ancestry, East Asian, and European GWAS are publicly available at the NHGRI-EBI GWAS Catalog (https://www.ebi.ac.uk/gwas/downloads) with accession numbers GCST90319904, GCST90319905, and GCST90319906, repectively. The UKBB genotype and epidemiologic data are available by requesting access on the UKBB homepage (https://www.ukbiobank.ac.uk/). Summary statistics are publicly available from the Chronic Kidney Disease Genetics Consortium (CKDGen, http://ckdgen.imbi.uni-freiburg.de/). BBJ summary statistics were downloaded from the Biobank Japan PheWeb (https://pheweb.jp/). The full summary statistics of the KBA GWAS are available at the NHGRI-EBI GWAS Catalog (https://www.ebi.ac.uk/gwas/downloads) and the Korea National Institute of Health PheWeb (https://coda.nih.go.kr/usab/pheweb/intro.do). The GTEx data are publicly available upon reasonable application (http://www.gtexportal.org/home/datasets). The NEPTUNE eQTL data are publicly available (https://nephqtl.org). The HERMES GWAS summary statistics are publicly available (https://www.hermesconsortium.org). The FinnGen GWAS summary statistics are publicly available (https://www.finngen.fi/en). The GUGC GWAS summary statistics are publicly available (https://kp4cd.org/node/179).

Research involving human participants, their data, or biological material

Policy information about studies with human participants or human data. See also policy information about sex, gender (identity/presentation), and sexual orientation and race, ethnicity and racism.

Reporting on sex and gender	We used sex as a covariate to adjust our analyses. In total, 482,170 males (46.84%) and 547,153 females (53.16%) were included in the cross-ancestry meta-analysis. There is no mention of gender in this study.
Reporting on race, ethnicity, or other socially relevant groupings	A GWAS was conducted for a total of 1,029,323 individuals of cross-ancestry. European and East Asian ancestry GWAS included 677,373 and 219,768 individuals, respectively.
Population characteristics	Analyses used age, sex, and principal components from the genetic data as covariates to control for the population structure. More population characteristics are described in Supplementary Table 51.
Recruitment	Researchers in this study were not involved in the participant recruitment.
Ethics oversight	This study was conducted using bioresources from the National Biobank of Korea, the Korea Disease Control and Prevention Agency, Republic of Korea (NBK-2019-063). The UK Biobank has obtained ethical approval covering this study from the National Research Ethics Committee (REC reference 11/NW/0382).

Note that full information on the approval of the study protocol must also be provided in the manuscript.

Field-specific reporting

Please select the one below that is the best fit for your research. If you are not sure, read the appropriate sections before making your selection.

☒ Life sciences ☐ Behavioural & social sciences ☐ Ecological, evolutionary & environmental sciences

For a reference copy of the document with all sections, see nature.com/documents/nr-reporting-summary-flat.pdf

Life sciences study design

All studies must disclose on these points even when the disclosure is negative.

Sample size	The total sample size was 1,029,323 individuals of cross-ancestry, consisting of 482,170 males (46.84%) and 547,153 females (53.16%). We

Sample size	tried to analyze as many samples as possible rather than statistica sample size calculation. The total sample size of the current study was the largest sample size to date.
Data exclusions	We followed elaborate quality control procedures to exclude all ineligible samples and variants. Detailed information is described in the Methods section.
Replication	This study was the largest cross-ancestry GWAS for serum urate in each ancestry. As a result, there was no external data to direct replication for GWAS results. For the polygenic risk score, 22,607 individuals from KoGES cohort, who were independent of the discovery samples, were included in the validation.
Randomization	Because this study is a meta-analysis of GWAS results from multiple cohorts, it is not a clinical experimental study, and randomization was not necessary at the discovery stage. Additionally, we aimed to identify genetic variants associated with serum urate by adjusting for age, sex, and principal components of genetic ancestry instead of randomization in our analyses.
Blinding	Because this study is a meta-analysis of GWAS results from multiple cohorts, it is not a clinical experimental study, and blinding was not necessary.

Reporting for specific materials, systems and methods

We require information from authors about some types of materials, experimental systems and methods used in many studies. Here, indicate whether each material, system or method listed is relevant to your study. If you are not sure if a list item applies to your research, read the appropriate section before selecting a response.

Materials & experimental systems

n/a	Involved in the study
☒	☐ Antibodies
☒	☐ Eukaryotic cell lines
☒	☐ Palaeontology and archaeology
☒	☐ Animals and other organisms
☒	☐ Clinical data
☒	☐ Dual use research of concern
☒	☐ Plants

Methods

n/a	Involved in the study
☒	☐ ChIP-seq
☒	☐ Flow cytometry
☒	☐ MRI-based neuroimaging

Plants

Seed stocks	*Report on the source of all seed stocks or other plant material used. If applicable, state the seed stock centre and catalogue number. If plant specimens were collected from the field, describe the collection location, date and sampling procedures.*
Novel plant genotypes	*Describe the methods by which all novel plant genotypes were produced. This includes those generated by transgenic approaches, gene editing, chemical/radiation-based mutagenesis and hybridization. For transgenic lines, describe the transformation method, the number of independent lines analyzed and the generation upon which experiments were performed. For gene-edited lines, describe the editor used, the endogenous sequence targeted for editing, the targeting guide RNA sequence (if applicable) and how the editor was applied.*
Authentication	*Describe any authentication procedures for each seed stock used or novel genotype generated. Describe any experiments used to assess the effect of a mutation and, where applicable, how potential secondary effects (e.g. second site T-DNA insertions, mosiacism, off-target gene editing) were examined.*
